# Evaluation of health status in patients with hepatitis c treated with and without interferon

**DOI:** 10.1186/s12955-018-0842-x

**Published:** 2018-01-17

**Authors:** R. Ragusa, G. Bertino, A. Bruno, E. Frazzetto, F. Cicciu, G. Giorgianni, L. Lupo

**Affiliations:** 1 Health Technology Assessment Committee, University Hospital “G. Rodolico”, Via Rosso di San Secondo 3, 95128 Catania, Italy; 2 Hepatology Unit-Department of clinical and Experimental Medicine, University Hospital “G. Rodolico”, Catania, Italy; 3 Science of Health Professions Technical Diagnostic, University of Catania, Catania, Italy; 4 School of Specialization in Internal Medicine, University of Catania, Catania, Italy; 5 School of Specialization in Hygiene, University of Catania, Catania, Italy; 6 Medical Statistic - Department of Medical and Surgical Sciences and advanced technologies, University of Catania, Catania, Italy

**Keywords:** Direct-acting antiviral agents (DAAs), Quality of life (QoL), Interferon (IFN), Hepatitis C, EQ-5D-5 L, EQIndex, Visual analog scale, Questionnaire

## Abstract

**Background:**

The evolution of technology in healthcare has increased the health care’s costs and, the universal healthcare systems, in developed countries, need to ensure proper allocation of resources. Thus, the major issue is assessing the effectiveness of new medical technologies. The evaluation of quality of life in response to new treatments has become a key indicator in chronic conditions for which medical interventions are evaluated not only in terms of increasing the number of expected life years but also in terms of increasing quality of life. The aim of this observational study was to verify whether a simple instrument (EQ-5D-5 L) can capture variations in health-related quality of life (HRQoL) and allow us to evaluate the impact of different drug treatment protocols in patients with hepatitis C virus (HCV) on daily activities.

**Methods:**

Sixty six patients with HCV were consecutively enrolled in the Hepatology Unit at the University Hospital of Catania “*G. Rodolico*”. Sixteen patients received new direct-acting-antiviral agents (DAAs) plus pegylated alpha interferon (Peg-α-IFN) protocol (Group A) and 50 DAAs IFN free protocol (Group B).

The EQ-5D-5 L® questionnaire and visual analog scale (VAS) were given to both groups to calculate coefficient’s utility. We used the EQ-5D-5 L Crosswalk Index Value Calculator to obtain the utility EQIndex and both parametric and non parametric tests for the statistical analysis.

**Results:**

The biopsy taken at the beginning of treatment showed comparable cell damage in both groups. The difference in the VAS results was negative for patients who received protocols containing IFN (indicating decreased quality of life),whereas it was positive in patients treated with IFN-free protocols. The baseline EQIndex did not reveal any differences between the two treatment groups. The post-treatment EQIndex was statistically better in the groups that received IFN-free therapy.

**Conclusions:**

When innovative treatments are introduced into clinical practice, assessing quality of life is mandatory to determine their benefits. The instruments used in the present study are effective in detecting the areas in which improvement has occurred. These instruments can be easily managed by general practitioners for follow up of progression of the disease and referred to the specialist.

## Background

The evolution of technology has increased the health care’s costs and the universal healthcare systems need to ensure proper allocation of resources. Thus, the major issue is assessing the effectiveness of new medical technologies [1, 2]. Benefits must be measured by criteria other than survival (efficacy) alone. In particular, in chronic conditions improvement in quality of life (QoL) has become a valuable indicator for determining the costs and benefits of new treatments [3–6]. Health-related QoL (HRQoL) is considered to represent the set of qualitative aspects of an individual’s life that are related to the domains of health and disease and therefore modifiable by medicine [7].

The EQ-5D is a standardized measure of health status that provides a simple measure of health for clinical and economical appraisal [8–12]. It is composed of a questionnaire and a visual analog scale (VAS), which are used to calculate utility coefficients (EQ index). The 5D-5 L is the latest version used in cost-utility analyses and is better than the previously used EQ-3D-3 L or EQ-5D-3 L [13, 14]. Quality-adjusted life years (QALYs) can be determined by the EQ index for one-year intervals. A QALY of 1 corresponds to a life expectancy of one year in good health; a value of 0 corresponds to death.

The EQ index can also represent HRQoL, which includes multidimensional functional, subjective, emotional and overall well-being indicators of an individual’s overall health status. The latter has become an important outcome measure in the evaluation of chronic disease management over the past 20 years [15–18].

The aim of this work was to assess the effect of two different drug treatment protocols (new direct-acting antiviral agents) with and without interferon (IFN) on QoL in patients with hepatitis C through EQ index calculations. We intend to evaluate the ability of this simple but non-specific instrument to detect the differences in clinical criteria that are due to the different treatments and the feasibility of utilizing this tool in clinical practice.

## Methods

### Subjects and setting

Sixty six patients who were diagnosed HCV disease eligible for treatment, by regional referral center at the Hepatology Unit of the University Hospital “*G. Rodolico*” in Catania, were consecutively enrolled from May 2014 to January 2016.

Sixteen of them were treated with DAAs plus Peg- α- IFN (Group A) and 50 received DAAs IFN free Protocol (Group B). Both groups were administered Eq-5D-5 L questionnaire before (subgroup1) and at the end of the treatment (subgroup 2).

Informed consent was previously obtained by each patient.

For inclusion in this study, patients had to be adults over 18 years old with a history of viral chronic liver disease (CLD) due to HCV and with no history of any of the clinical features of encephalopathy. All patients had chronic fibrosis (level III-IV) at biopsy.

Patients with mental disorders or dementia were excluded. Individuals with concomitant comorbidities such as heart failure, chronic renal failure, chronic obstructive pulmonary disease, malignancy and inflammatory bowel disease (ulcerative colitis and Crohn’s disease) were also excluded.

Patients followed two different protocols of treatment with direct-acting antiviral agents due to the different timing of marketed products. Until October 2015, patients were consecutively treated with direct-acting agents plus IFN. Afterward, new IFN-free direct-acting drugs became available in Italy. Sixteen patients (group A) were treated with Boceprevir or Telepravir plus Peg-α-IFN and ribavirin. Fifty patients (group B) were treated with the new IFN-free direct-acting antiviral agent (DAAs) protocols (Sofosbuvir, Simeprevir, Ombitosvir/ Paritaprevir/ Ritonavir + Dasabuvir, Daclatasvir) for 12 weeks. Seven patients in group A received IFN + Boceprevir and 9 received IFN + Telaprevir. Ten patients in group B received Sovaldi (Sofosbuvir + Ribavirin; 31 received Olisyio (Simeprevir + Sofosbuvir), 5 received Viekirax + Exviera) and 4 received Daklinza (Daclatasvir).

We administered the EQ-5D-5 L questionnaire and VAS to both groups during a face-to-face interview, before and three months after the end of treatment. The EQ-5D-5 L questionnaire was administered by two different referrals in a waiting room inside the clinic and was administered in the patient’s native language.

### Instruments

EQ-5D is a standardized instrument developed by EuroQol Group that is used to measure a patient’s health status as a single index value [19]. The EQ-5D-5 L version consists of 2 pages: the EQ-5D descriptive system and the EQ-VAS.

**Fig. 1:**
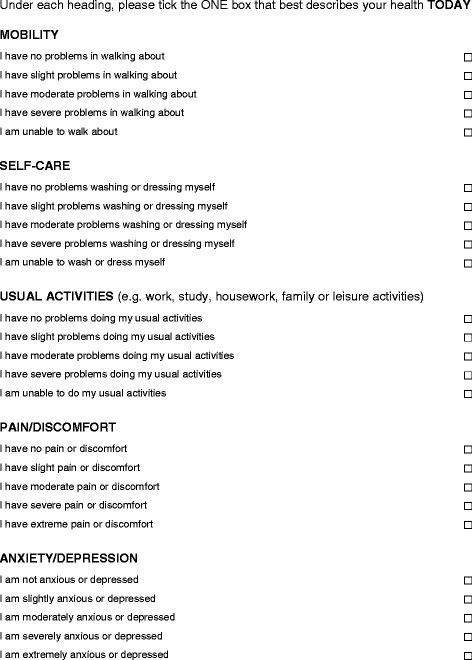
EQ-5D-5 L Questionnaire (UK sample version)

The EQ-5D-5 L descriptive system is a self-administered standardized questionnaire that includes five dimensions, each with5 levels (Fig. 1). The EQ-5D-5 L is an indirect method for measuring a patient’s health status. The EQ-5D-5 L descriptive system comprises the following dimensions: mobility, self-care, usual activities, pain/discomfort and anxiety/depression. Each dimension has the following 5 levels: no problems, slight problems, moderate problems, severe problems and extreme problems. The respondent is asked to indicate his/her health status by ticking (or placing a cross in) the box next to the most appropriate statement in each of the 5 dimensions. In the EQ-5D-5 L, as each dimension has five levels [1 to 5], scores range from 1-11-1-1 (best health) to 5-55-5-5 (worst health).

**Fig. 2:**
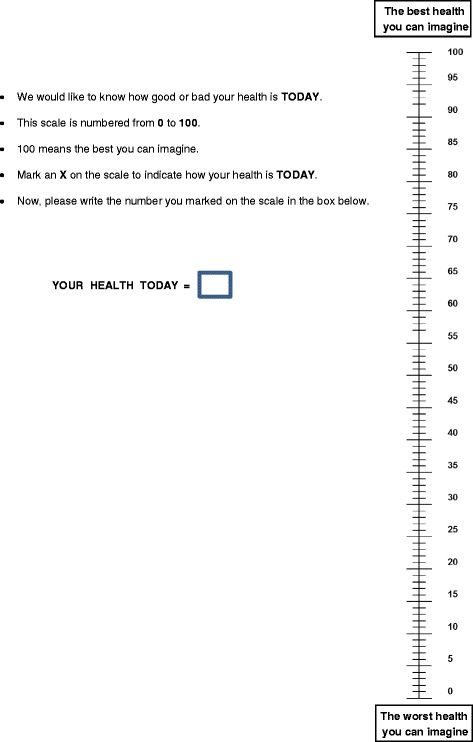
Visual Analogue scale (VAS)

The EQ-VAS (Fig. 2) is also an integral part of the EQ-5D-5 L. The VAS is a continuous scale on which a cross along the line is directly marked by the patient according to his/her perceived health status. The VAS is a thermograph-like 20-cm linear scale rated from 0 (the worst imaginable health) to 1 (the best imaginable health). Subjects are asked to mark the scale according to their current health status, and the fraction to unity is reported. This information can be used as a quantitative measure of health outcome as judged by the individual respondents.

Both instruments (the Eq-5D-5 L descriptive system and EQ-VAS) are combined to obtain the utility EQ index for each subject using the crosswalk link function [20].

### Analysis

There are country-specific combinations of either EQ-5D-5 L datasets or reported VAS values into a single index value (EQ index). Since no specific Italian combination data sets exist, we decided to adopt the Spanish dataset, which was the closest available dataset. However, because we wanted to examine the differences within each subject before and after treatment, no bias can be attributed to the use of the Spanish dataset.

Using the crosswalk link function and the individual responses to the EQ-5D-5 L descriptive system, index values for the EQ-5D-5 L were calculated. Documents containing information on the crosswalk project, tables of values for all 3125 health statuses and the ‘*EQ-5D-5 LCrosswalk Index Value Calculator’* can be downloaded from the EuroQol website [21].

The mean values and standard deviations (SDs) of the differences in the VAS and EQ indexes of the two different treatment groups were calculated.

We used a paired Student’s t-test to compare the pre- and post-treatment paired VAS data between the two subgroups of group A (IFN treatment) and the two subgroups of group B (IFN-free treatment) using a 5% two-sided significance level.

An unpaired Student’s t-test was used to compare the EQIndex values obtained in the two groups at the end of treatment.

## Results

A total of 128 questionnaires were administered. The complete results refer to16 patients from group A and 46 patients from group B. Four patients from group B did not complete the second questionnaire. In group A, 68% of the patients were males and 32% were females, whereas in Group B 58% were males and 42% were females. The avearage age of patients was 59 years in both groups Table 1 shows the homogeneous distribution of the patients by age and group.

**Table 1:** Distribution patients by age and group

Age (years)	31–40	percent	41–50	percent	51–60	percent	61–70	percent	> 71	percent
Group A	0	*/*	3	19%	5	31%	6	37%	2	13%
Group B	3	*6%*	8	16%	16	33%	12	25%	11	22%

The average time taken to administer the test was 7 min. All of the patients understood the questions and quickly indicated an answer, suggesting that reporting the perceived health status as shown in the graph was simple and intuitive.

The data were later input into the program *‘EQ-5D-5 L Crosswalk Index Value Calculator’*, which had previously been downloaded from the EuroQol website.

Table 2 shows the replies for the 5 dimensions of the questionnaire in the two groups of patients who underwent the different therapies. The table shows the percentage of patients with NO problems (level 1) and the percentage of patients with no problems for each of the 5 dimensions of the questionnaire. The comparisons are made within each treatment and between the groups. The data are reported as the percentage of the total.

**Table 2:** Frequency of reporting of problems in both group of treatment

		Group A				Group B			
		PRE	*(%)*	POST	*(%)*	PRE	*(%)*	POST^a^	*(%)*
MOBILITY	NO Problem	10	*62*	10	*62*	29	*58%*	33	*72%*
	Problem	6	*38*	6	*38*	21	*42%*	13	*28%*
SELFCARE	NO Problem	13	*81*	11	*69*	46	*92%*	42	*91%*
	Problem	3	*19*	5	*31*	4	*8%*	4	*9%*
USUAL ACTIVITY	NO Problem	8	*50*	8	*50*	34	*68%*	37	*80%*
	Problem	8	*50*	8	*50*	16	*32%*	9	*20%*
PAIN/DISCOMFORT	NO Problem	7	*44*	6	*37*	27	*54%*	32	*70%*
	Problem	9	*56*	10	*63*	23	*46%*	14	*30%*
ANXIETY/DEPRESSION	NO Problem	7	*44*	2	*12*	35	*70%*	35	*76%*
	Problem	9	*56*	14	*88*	15	*30%*	11	*24%*

^a^Four patients did not complete the second questionnaire

The percentage of patients with NO problems decreased in three of the dimensions (self-care, pain/discomfort, and anxiety/depression) in group A, whereas the percentage of patients with NO problems increased in four of the dimensions (mobility, self-care, usual activity and pain/discomfort) in group B. The percentage of patients receiving IFN-free therapy (group B) who reported anxiety/depression remained unchanged, whereas the percentage of patients reporting anxiety/depression more than doubled in group A.

Table 3 and Table 4 show the frequencies of the five dimensions in the different groups.

**Table 3:** Frequencies of the five levels for each dimension in the Group A pre and post treatment

EQ-5D DIMENSION	LEVEL 1 (%)		LEVEL 2 (%)		LEVEL 3 (%)		LEVEL 4 (%)		LEVEL 5 (%)		PRE n.16
MOBILITY	10	*62*	4	*25*	2	*13*	0	/	0	*/*	
SELFCARE	13	*81*	3	*19*	0	*/*	0	/	0	*/*	
USUAL ACTIVITY	8	*50*	7	*44*	1	*6*	0	/	0	*/*	
PAIN/DISCOMFORT	7	*44*	4	*31*	5	*25*	0	/	0	*/*	
ANXIETY/DEPRESSION	7	*44*	6	*37*	3	*19*	0	/	0	*/*	
EQ-5D DIMENSION											
MOBILITY	10	*62*	4	*25*	2	*13*	0	*/*	0	*/*	POST n.16
SELFCARE	11	*69*	5	*31*	0	*/*	0	*/*	0	*/*	
USUAL ACTIVITY	8	*50*	5	*31*	3	*19*	0	*/*	0	*/*	
PAIN/DISCOMFORT	6	*37*	3	*19*	3	*19*	4	*25*	0	*/*	
ANXIETY/DEPRESSION	2	*13*	8	*50*	5	*31*	0	*/*	1	*6*

**Table 4:** Frequencies of the five levels for each dimension in the Group B pre and post treatment

EQ-5D DIMENSION	LEVEL 1 (%)		LEVEL 2 (%)		LEVEL 3 (%)		LEVEL 4 (%)		LEVEL 5 (%)		PRE n.50
MOBILITY	29	*58%*	12	*24%*	5	*10%*	3	*6%*	1	*2%*	
SELFCARE	46	*92%*	1	*2%*	2	*4%*	0	*0%*	1	*2%*	
USUAL ACTIVITY	34	*68%*	10	*20%*	4	*8%*	1	*2%*	1	*2%*	
PAIN/DISCOMFORT	27	*54%*	17	*34%*	2	*4%*	3	*6%*	1	*2%*	
ANXIETY/DEPRESSION	35	*70%*	11	*22%*	1	*2%*	3	*6%*	0	*0%*	
EQ-5D DIMENSION											
MOBILITY	33	*72%*	5	*11%*	7	*15%*	0	*0%*	1	*2%*	POST n.46
SELFCARE	42	*91%*	2	*5%*	1	*2%*	0	*0%*	1	*2%*	
USUAL ACTIVITY	37	*80%*	4	*9%*	3	*7%*	1	*2%*	1	*2%*	
PAIN/DISCOMFORT	32	*70%*	7	*15%*	6	*13%*	1	*2%*	0	*0%*	
ANXIETY/DEPRESSION	35	*76%*	8	*18%*	2	*4%*	1	*2%*	0	*0%*	

The descriptive statistics, including the means, standard deviations and ranges of the EQ-VAS analyses in the two groups pre- and post-treatment are presented in Table 5. We used a paired Student’s t-test to compare the pre- and post-treatment paired VAS data between the two subgroups of group A (IFN treatment) and the two subgroups of group B (IFN-free treatment). By comparing the mean of the differences before and after treatment in group A, we found no significant difference (t_n-1_ 1,64). By comparing the mean of the differences before and after treatment in group B, we found a significant difference (t_n-1_ 3816) (*P* < 0.01).

**Table 5:** Values of EQ-VAS analysis in the two groups pre and post treatment

EQ VAS	Group A		Group B	
	PRE	POST	PRE	POST
Mean	66.6	62.2	74.1	80.1
SD	15	12.8	19.9	17
Median	67.5	60	80	80
25th	50	50	60	71.25
75th	76.25	72.5	90	90
t test	(*P* > 0.05)		(*P* < 0.001)	
sign test	(*P* > 0.05)		(*P* > 0.001)	

A non parametric sign test was also performed in order to evaluate the direction of the differences in the VAS score of each patient before and after treatment both in Group A and Group B.

While there was no significant differences between those who gave a better answer in VAS score after treatment in group A, we found highly significant difference in those who reached a higher VAS score (45.6%) after treatment in Group B, while only 6.5% signed a worse score.

The EQ Index for each patient in both groups before and after treatment and the averages, standard deviations, medians, and 25th and 75th percentiles in the various groups were calculated. The descriptive statistics for the EQ Index analysis in the two groups pre- and post-treatment are shown in Table 6.

**Table 6:** Analysis of EQ INDEX in both groups pre and post treatment

EQ INDEX	Group A		Group B	
	PRE	POST	PRE	POST
Cases	16	16	46	46
Mean	0.82	0.71	08.1	0.88
SD	0.13	0.22	0.29	0.25
Median	0.85	0.72	0.91	0.97
25th	0.73	0.56	0.79	0.86
75th	0.9	0.89	1	1
EQ Index Post treatment				
T _n-2_	2.40 (*P* < 0,02)			

The average EQ Index before treatment was 0.82 in group A and 0.81 in group B. There was no significant difference. We used an unpaired Student’s t-test to compare the post-treatment EQIndex values between groups A and B, and the t_n-2_ value obtained was 2.40 (*P* < 0.02).

The difference between the means of the EQ INDEX values before and after treatment in Group A was - 0.111; the difference between the means of the EQ INDEX values before and after treatment in Group B was 0.062. The averages of the differences obtained in the group A and the group B showed a statistically significant difference (*P* < 0.0001).

## Discussion

The availability of DAAs enables highly efficacious treatment of chronic HCV infection [22]; however, whenever a new drug is approved, particularly if it is a high-impact therapy, an economic assessments should be performed to determine whether the medication will produce real health benefits for patients and cost savings for the healthcare system [23–26].

Previous studies on the HRQoL of patients treated for HCV infection have used specific questionnaires [27–30]. We wanted to determine whether a non-specific questionnaire could provide QoL data in patients with the same disease who are treated with different regimens.

In patients with liver damage caused by HCV, once the viral infection is eradicated, the mode and duration of patient monitoring are determined by the degree of impairment of the liver before treatment. Thus, if the patient was cirrhotic, he or she should continue the treatment protocol and be monitored for complications of liver cirrhosis for approximately 2 years, after which the HCV surveillance protocol will end.

The patients included in our study completed the questionnaire before beginning treatment and three months after the end of treatment to investigate decrements in everyday functional health status and general health perception.

In the group B (IFN-free treatment), the increase in the EQ-VAS after treatments with DAAs was statistically significant. The perceived QoL decreased in the patients treated with IFN while improved in patients treated with IFN-free therapy.

The EQ Index was comparable between the two groups of patients before treatment: the two treatment groups included patients with the same disease characteristics. There was no difference between the EQ index in the subjects who received Telaprevir and Boceprevir. In addition, there was no difference between the EQ index in the subjects who received Sofosbuvir and Simeprevir.

We compared the post-treatment values, and the values for the patients who received IFN treatment were significantly lower than those for the patients who received IFN-free treatment.

Therefore, the EQ Index in the group that received IFN-free treatment was significantly higher than that of the group that received IFN treatment.

IFN-free treatment guarantees a better QoL instead of the baseline condition. Compared with IFN treatment, IFN-free treatment significantly improves the EQIndex.

This combined approach (EQ-VAS plus EuroQol 5D) could be a useful tool for monitoring the health of patients. The instrument, although generic, has been shown in our study to be simple, fast and capable of determining changes in the health of patients. This instrument can also be easily managed by general practitioners for follow up of progression of the disease and referred to specialist.

The Italian Association for the Study of the Liver (AISF) published a joint document with general practitioners for the appropriate management of patients with HCV, in which the term “management” includes the identification of risk factors and risk behaviors, as well as the clinical circumstances in which the diagnosis of HCV should be suspected [31].

Patients without access to new DAAs therapies should be monitored for the progression of the disease by both the specialist and doctor.

The role of the general practitioner remains important, even after the eradication of the infection [32, 33]. In addition to the first few months post-therapy, during which the patient will remain under the supervision of the specialist, it is important that the physician ensures the subsequent monitoring of the disease, particularly for patients with advanced disease who remain at risk of developing liver disease complications despite undergoing treatment for HCV [34–37].

Even monitoring changes in any of the dimensions of EQ INDEX may reveal impairment of health status and subsequent progression of the disease to be referred to hepatologist.

## Conclusions

There was no difference in the average EQ Index between the two groups before treatment. The post-treatment EQ Index was statistically better in the group that received IFN-free therapy (group B). There was none evidence of a significant difference between the two treatment groups; the only difference was highlighted by the presence of IFN.

This type of combined approach (EQ-VAS plus EuroQol 5D) could be a useful tool for monitoring the health of patients. This tool has proven to be simple, fast and capable of detecting changes in the health of patients. The EQ-5D-5 L has also shown some sensitivity for detecting small variations, which, if systematically collected and thoroughly analyzed by the program [20] can be useful to monitor the effects of different treatments.

The EQ-5D-5 L can be even filled by patient himself and sent to the general practitioner or hepatologist, allowing a proper follow-up of the patient without the necessity of having the patient visit the reference center. The instrument is a particularly useful tool to follow-up patients who can not be treated immediately and is a useful surveillance tool for specialists.

The data in this study suggest that the main cause of the reduction in the VAS score and EQIndex in our patients was IFN therapy. Follow up of their values along with deterioration of the items in the EQ-5D-5 L can be predictive of relapse.

## Abbreviations

AISF: Italian Association for the Study of the Liver
CLD: Chronic liver disease
DAAs: Direct-acting antiviral agents
EQ-5D-5 L: EuroQol standard questionnaire with five dimensions, each with5 levels
HCV: Hepatitis C virus
HRQoL: Health-related quality of life
IFN: Interferon
NHS: National Health System
Peg-α-IFN: Pegylated alpha interferon
QALY: Quality adjusted life year
QoL: Quality of life
SD: Standard deviation
VAS: Visual analog scale
